# A pan-cancer analysis of STAT3 expression and genetic alterations in human tumors

**DOI:** 10.1515/med-2023-0792

**Published:** 2023-09-16

**Authors:** Junyin Tan, Ronghao Feng

**Affiliations:** Department of Oncology, Guigang People’s Hospital of Guangxi/The Eighth Affiliated Hospital of Guangxi Medical University, Guigang, Guangxi, China

**Keywords:** pan-cancer, STAT3, prognosis, immune microenvironment, genetic alteration

## Abstract

Combined cancer immunotherapy and targeted therapy have proven to be effective against various cancers and therefore have recently become the focus of cancer research. Signal transducer and activator of transcription 3 (STAT3) is a member of the STAT protein family of transcription factors. Several studies have shown that STAT3 can affect the prognosis of cancer patients by regulating immune microenvironment (IME). Therefore, STAT3 may have high research value for the development of combined immunotherapy/targeted therapy approaches for the treatment of cancer patients. We found differences in STAT3 expression between tumor and normal tissues. Kaplan−Meier survival and Cox regression analyses showed that high expression of STAT3 is associated with poor prognosis in low-grade glioma (LGG) patients. The results of the analysis of the area under the curve of the receiver operating characteristic curve further suggested that the expression of STAT3 is an effective way to evaluate the prognosis of patients with glioma. The results of the IME analysis revealed that the immune and matrix scores of LGGs were positively correlated with the expression of STAT3 (*P* < 0.05). The results of immune cell infiltration analysis showed that STAT3 was positively correlated with resting dendritic cells, eosinophils, neutrophils, M0 macrophages, M1 macrophages, CD4 memory resting T cells, and CD8 T cells in LGG patients, but negatively correlated with activated mast cells and M2 macrophages (*P* < 0.05). Our gene set enrichment analysis identified 384 enriched pathways. According to the enrichment scores, the top ten most significantly upregulated pathways were related to immune response. The top ten most significantly downregulated pathways were related to cell signal transduction and the regulation of cell survival, proliferation, and metabolism. Genetic alteration analysis showed that missense mutations in STAT3 account for the majority of mutations, and STAT3 mutations mostly occur in the Src homology domain. In conclusion overexpression of STAT3 can promote the development and growth of tumors by regulating IME, which is significantly related to the poor prognosis of cancer patients. Therefore, targeted inhibition of STAT3 expression may have high research value for the development of combined immunotherapy/targeted therapy approaches for the treatment of cancer patients.

## 1 Introduction

Combined immunotherapy and targeted therapy have become a hot topic in cancer research, and immune therapy targeting immunosuppressive genes, such as CD274, PDCD1, CTLA4, LAG3, TIGIT, etc., have been shown to be effective. However, many clinical and experimental studies have revealed that not all tumors respond to immunotherapy against these targets. Therefore, finding new potential targets is critical for the development of more effective cancer therapies.

The signal transducer and activator of transcription (STAT) protein family of transcription factors in mammals consists of seven members (STAT1, STAT2, STAT3, STAT4, STAT5A, STAT5B, and STAT6), which are associated with the regulation of the cell cycle, cell survival, and immune response [1−11]. STAT3, which was first reported by Akira et al. [12], is a protein composed of 770 amino acids with six functionally conserved domains, including the amino-terminal domain, coiled-coil domain, DNA-binding domain, linker domain, Src homology 2 (SH2) domain, and trans-activation domain [13]. In most cancers, STAT3 is overactivated and promotes tumor progression by regulating various biological processes, such as proliferation, apoptosis, angiogenesis, and immune response, which are generally associated with poor clinical prognosis [10,14−17]. Therefore, targeting the STAT3 signaling pathway has been recognized as a promising therapeutic strategy for numerous cancers [5].

Additionally, many studies have shown that STAT3 can affect the prognosis of tumor patients by regulating the immune microenvironment (IME) [18−20]. There are studies to prove that derivatives of secondary metabolites can play an anticancer effect by regulating the STAT3 pathway, and show cytotoxicity to cancer cells but no toxicity to non-cancer cell lines, which may inspire development of new drug-like substances with improved cytotoxicity on cancer [21−23]. However, the specific molecular mechanisms of STAT3 in the pathogenesis of different tumors remain unclear, as is their value in the human pan-cancer analysis. In addition, through literature search, we have found few studies on pan-cancer analysis of STAT3 from the perspective of overall tumor microenvironment. Therefore, in this study, we mainly used bioinformatics methods to investigate the impact of changes in STAT3 expression and genetic alterations on the development of cancer from the pan-cancer perspective, to provide new insights into the transformation and application of STAT3 in the development of more effective cancer treatments.

## 2 Materials and methods

### 2.1 Data retrieval and pre-processing

In order to unify the standard, we obtained the gene expression data, clinical data, and sample information of 33 cancers from The Cancer Genome Atlas (TCGA) database from the University of California, Santa Cruz (UCSC) Xena database (http://xena.ucsc.edu/) [24]. Also, we downloaded mutation data from TCGA (https://portal.gdc.cancer.gov/repository) [25]. In addition, we obtained the annotation information of the genes from the Ensembl human genome browser GRCh38. P13 (http://asia.ensembl.org/index.html) [26]. Additionally, the data of 1,018 glioma samples were downloaded from the Chinese Glioma Genome Atlas (CGGA, http://www.cgga.org.cn/) and used for subsequent verification [27].

### 2.2 Gene expression analysis

Wilcoxon test analysis was performed to determine significant differences in STAT3 expression between cancer samples and normal samples. Additionally, we also analyzed the protein expression dataset obtained from The National Cancer Institute’s Clinical Proteomic Tumor Analysis Consortium (CPTAC) through the UALCAN website (http://ualcan.path.uab.edu/analysis-prot.html) [28]. To this end, we opened the UALCAN website and typed “STAT3” to obtain the total protein expression level of STAT3 between primary tumor and normal tissues.

### 2.3 Prognostic analysis

First, 33 types of cancer were divided into high and low expression groups according to the median level of expression of STAT3. The overall survival (OS) time and progression-free survival (PFS) of the high and low expression groups were analyzed by Kaplan−Meier (K−M) and Cox survival analyses using the “survival” package in R. The K−M survival curve and Cox forest plot were plotted with the “survminer” and “forestplot” packages, respectively. The dataset downloaded from the CGGA was used to verify the prognostic role of STAT3 in low-grade glioma (LGG). Additionally, we also performed univariate and multivariate prognostic analysis and receiver operating characteristic (ROC) curve analysis on the CGGA dataset to determine whether STAT3 expression can be an independent prognostic factor for glioma patients and its accuracy. The immunohistochemical staining data of STAT3 protein in normal and glioma tissues were obtained from the Human Protein Atlas (HPA, https://www.proteinatlas.org/).

### 2.4 Immune correlation analysis

Immunotherapy and IME have long been the focus of tumor research. In order to further understand the mechanism by which STAT3 affects cancer prognosis from the perspective of immunity, we performed immune correlation analysis on the cancers with statistical significance in the survival analysis described in the previous section. We first evaluated the IME of each tumor based on TCGA expression data using the “estimate” package in R to obtain an immune score and a stromal score for each tumor, followed by a “Spearman” correlation analysis between these scores and STAT3 expression. Then, we used the CIBERSORT algorithm to evaluate the degree of infiltration of 22 immune cells in each cancer type [29]. Subsequently, we calculated the correlation between the degree of infiltration of each immune cell and STAT3 expression using the “Spearman” test. We also performed immune checkpoint correlation analysis, determined the correlation between common immune checkpoints and STAT3 expression levels by “Spearman” correlation analysis, and visualized the results as a heat map.

### 2.5 Enrichment analysis

We performed gene set enrichment analysis (GSEA) to identify the pathways through which activated STAT3 promotes tumor development. To this end, we first downloaded the gene set database file “c2.cp.kegg.v7.1.symbols.gmt” from the “downloads” in the Molecular Signatures Database (MSigDB) in the GSEA website (http://www.gsea-msigdb.org/) [30]. The data of 33 cancer types of TCGA were divided into high and low expression groups according to the median level of expression of STAT3, and the data were downloaded from the “org.Hs.eg.db” “clusterProfiler” “enrichplot” R package for GSEA analysis, and a *P* value less than 0.05 was considered significant.

### 2.6 Genetic alteration analysis

According to the mutation data downloaded from TCGA, we determined the tumor mutational burden (TMB) and microsatellite instability (MSI) in each tumor. Then, we analyzed the correlation between the TMB and MSI for each tumor and the expression of STAT3, determined the correlation coefficient and *P*-value, and used the “fmsb” package in R to visualize the results as a correlation radar map. The CBioPortal database (http://www.cbioportal.org/) was used to obtain, visualize, and analyze multidimensional cancer genomic data for subsequent analysis of STAT3 gene alterations [31,32]. We selected “Pan-cancer analysis of whole genomes (ICGC/TCGA, Nature 2020)” in the “Query” module, clicked the “Query by gene” button, and entered the “STAT3” gene. The results of the structural variation data, mutation data, and CNA data are shown in the “Cancer Types Summary” module. The analysis results of STAT3 mutation and its three-dimensional (3D) structure are in the “Mutation” module. The Catalogue of Somatic Mutations in Cancer (COSMIC, http://www.sanger.ac.uk/cosmic) is a database that preserves somatic mutation data and related information for further analysis of STAT3 mutations [33]. We typed “STAT3” in the query module and clicked “SEARCH”. In the results, the Gene view, Tissue distribution, Variants, Mutation distribution, and 3D structure of the STAT3 gene can be easily seen by clicking “STAT3”.

## 3 Results

### 3.1 Gene expression analysis

**Figure 1:**
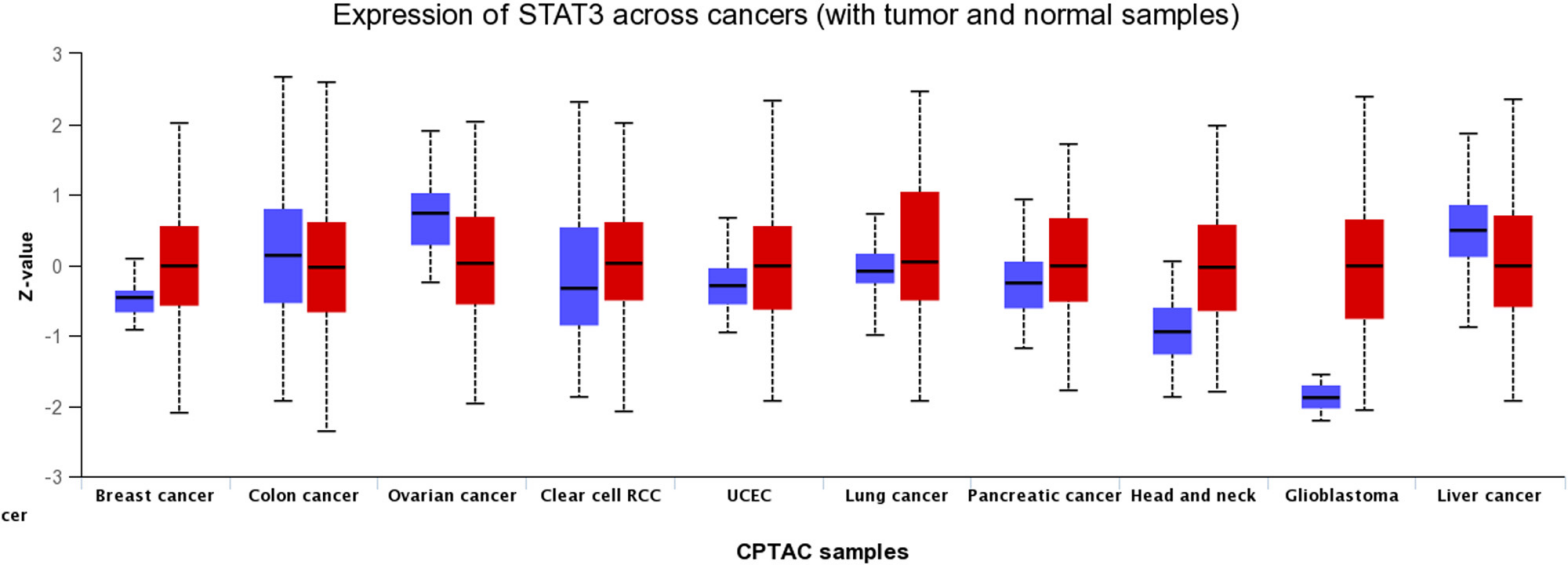
Gene expression analysis between normal and tumor tissues that are curated by UALCAN website (*P* < 0.005).

Ten types of tumors, namely breast cancer (BRCA), ovarian cancer, colon cancer, clear cell renal cell carcinoma (ccRCC), uterine corpus endometrial carcinoma (UCEC), lung cancer, head and neck squamous cell carcinoma (HNSC), glioblastoma, liver cancer, and pancreatic cancer in the CPTAC dataset were analyzed using the UALCAN web tool. The analysis revealed significant differences in STAT3 expression between the different types of tumors and normal tissues (*P* < 0.05, Figure 1). STAT3 was highly expressed in BRCA, ccRCC, UCEC, lung cancer, HNSC, pancreatic cancer, and glioblastoma, but lowly expressed in ovarian cancer, colon cancer, and liver cancer.

### 3.2 Prognostic analysis

**Figure 2:**
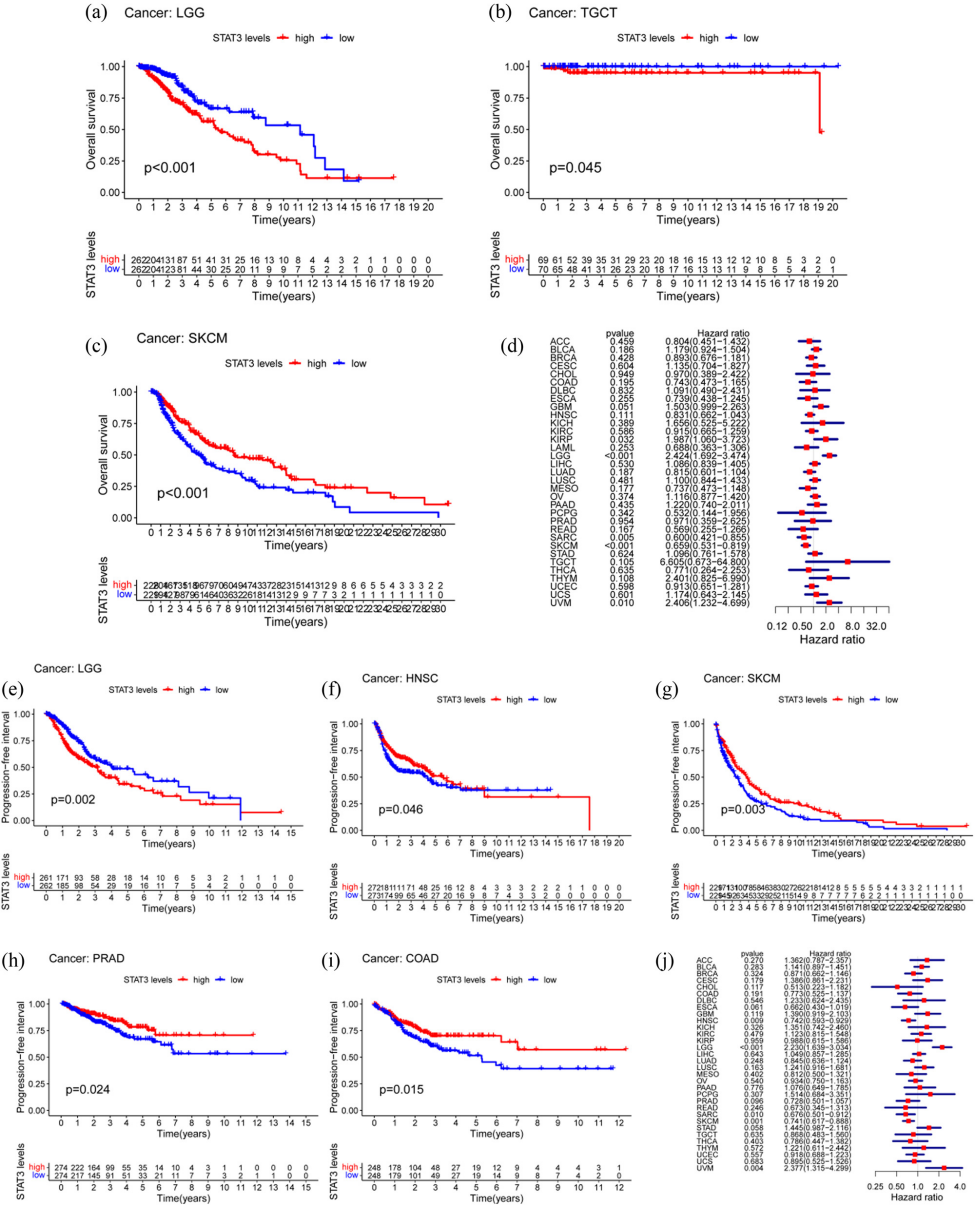
Prognostic survival analysis. (a)−(c) K−M survival analysis showed that the OS of LGG, TGCT, and SKCM in STAT3 high expression group was significantly different. (d) COX regression analysis showed that the OS of LGG and SKCM were significantly correlated with the expression of STAT3. (e)−(i) PFS of LGG, HNSC, SKCM, PRAD, and COAD were significantly different. (j) COX regression analysis showed that only LGG had significant difference in PFS and STAT3 expression.

**Figure 3:**
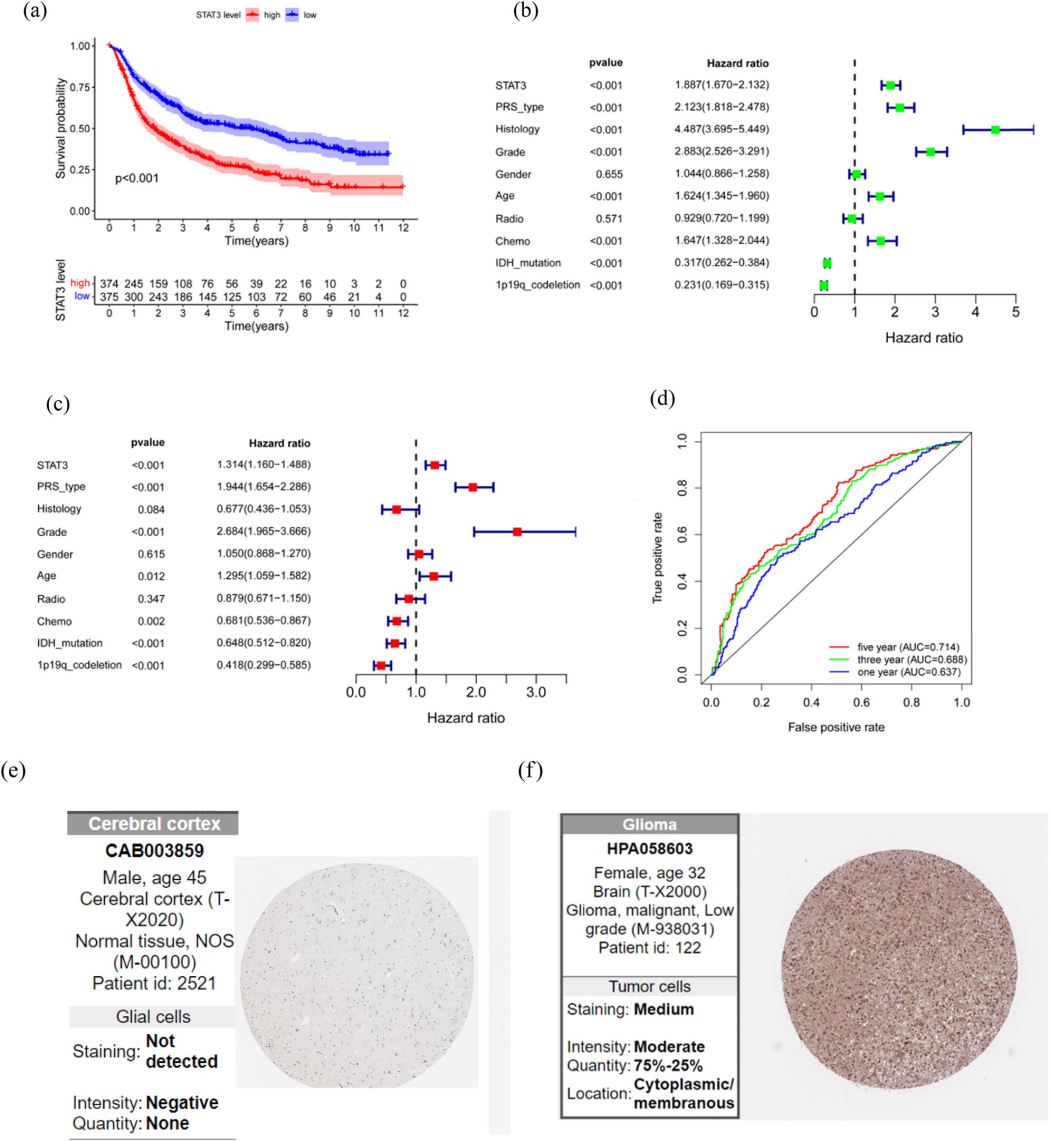
Verification of CGGA: (a) result of K−M survival analysis, (b) results of univariate analysis, (c) results of multivariate analysis, (d) ROC curve, (e) immunohistochemical staining showed that STAT3 was negative staining in normal tissue, and (f) immunohistochemical staining showed that STAT3 was medium positive in LGG.

We first performed the K−M survival and Cox regression analyses of TCGA data. The K−M survival analysis revealed that the OS of patients with LGG (Figure 2a), tenosynovial giant cell tumors (TGCT) (Figure 2b), and skin cutaneous melanoma (SKCM) (Figure 2c) in the STAT3 high expression group was significantly different (*P* < 0.05). In particular, in LGG and TGCT patients, the OS in the low expression group was higher than that in the high expression group, while in SKCM patients the OS in the high expression group was higher than that in the low expression group. Cox regression analysis revealed that the OS of patients with LGG and SKCM (Figure 2d) was significantly correlated with the expression of STAT3 (LGG: hazard ratio [HR] 2.424, ranging from 1.692 to 3.474, *P* < 0.001; SKCM: HR 0.659, ranging from 0.531 to 0.819, *P* < 0.001). The PFS of patients with LGG (Figure 2e), HNSC (Figure 2f), SKCM (Figure 2g), prostate adenocarcinoma (PRAD) (Figure 2h), and colon adenocarcinoma (COAD) (Figure 2i) was significantly different, and the PFS of the low STAT3 expression group was significantly higher than that of the high expression group in LGG. Cox regression analysis (Figure 2j) revealed that only patients with LGG had significant difference in PFS and STAT3 expression (HR 2.230, ranging from 1.639 to 3.034, *P* < 0.001). Thus, it is evident that the expression of STAT3 in LGG is correlated with OS and PFS, and the difference is statistically significant. In addition, we validated the results with the CGGA glioma dataset and OS, and found that the results were similar to those mentioned above (K−M: *P* < 0.001, Figure 3a; Cox: HR 1.887, ranging from 1.670 to 2.132, *P* < 0.001, Table 1). The results of univariate (U) and multivariate (M) analysis showed that the change in STAT3 expression was statistically significant in evaluating the prognosis of patients with LGG (U: HR 1.887, ranging from 1.670 to 2.132, *P* < 0.001, Figure 3b; M: HR 1.314, ranging from 1.160 to 1.488, *P* < 0.001, Figure 3c). The area under curve results obtained by ROC curve analysis (1 year: 0.637, 3 years: 0.688, and 5 years: 0.714, Figure 3d) further indicated that measuring the expression of STAT3 is an effective way to evaluate the prognosis of patients with glioma. Moreover, we examined the STAT3 protein expression levels in LGG and normal tissues obtained from the HPA database, and the immunohistochemical staining images showed negative immunostaining of STAT3 in normal tissue (Figure 3e) and moderately positive immunostaining in LGG (Figure 3f).

**Table 1:** Result of COX regression analysis in CGGA

Cancer	LGG
HR	1.887
HR.95L	1.670
HR.95H	2.132
*P* value	<0.001

### 3.3 Immune correlation analysis

**Figure 4:**
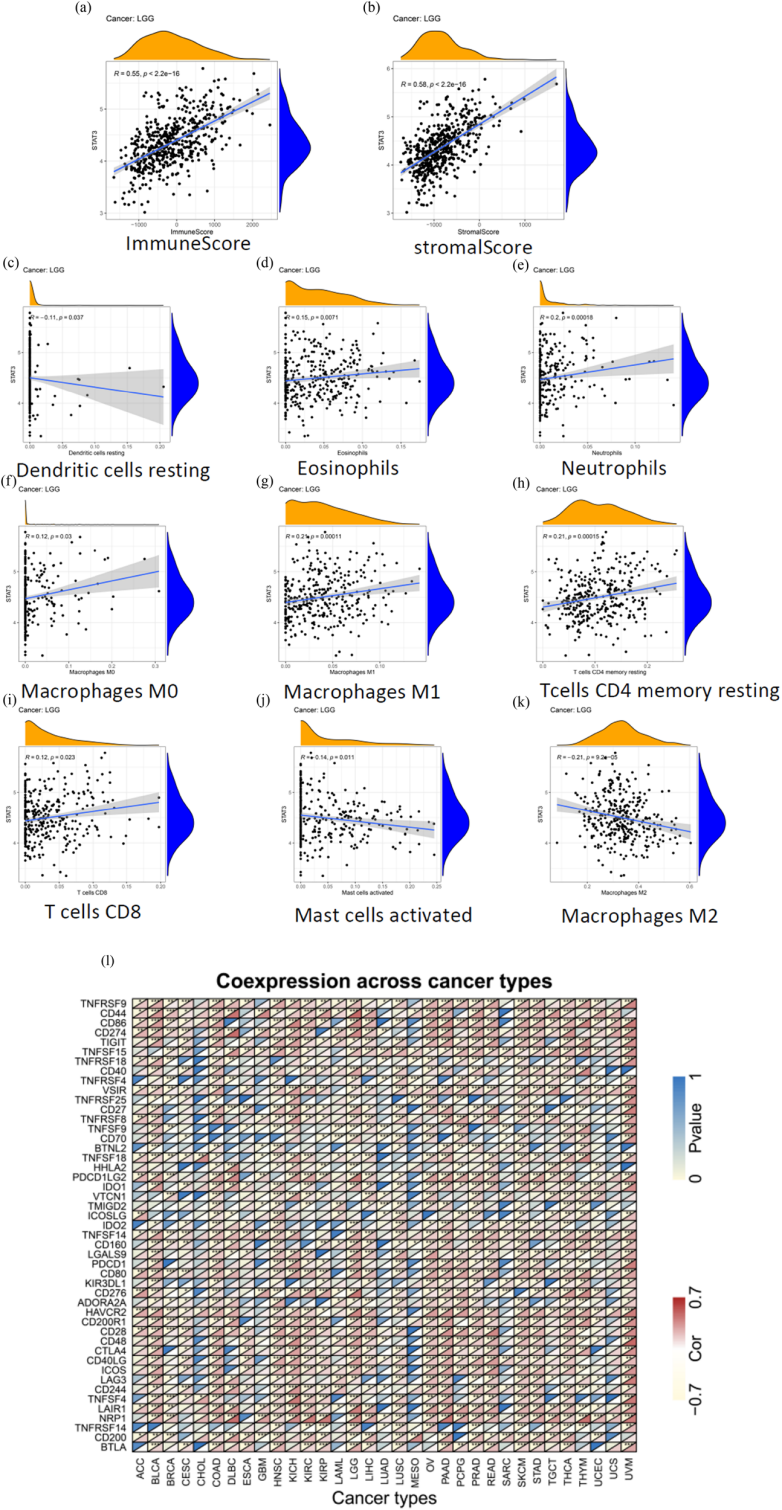
Results of immune correlation analysis: (a) immune score of LGG by IME analysis, (b) matrix score of LGG by IME analysis, (c)−(k) results of immune cell infiltration, and (l) correlations between immune checkpoints and STAT3 expression.

The survival analysis revealed that the expression of STAT3 in LGG is correlated with OS and PFS, and the difference is statistically significant. Therefore, we took LGG as an example for immune correlation analysis to further investigate the relationship between STAT3 expression and tumor immunity and the mechanism by which STAT3 affects prognosis of LGG patients. The results of the IME analysis revealed that the immune score (Figure 4a) and matrix score (Figure 4b) of LGG were positively correlated with the expression levels of STAT3 (*P* < 0.05). The results of immune cell infiltration showed that STAT3 was positively correlated with resting dendritic cells (DCs) (Figure 4c), eosinophils (Figure 4d), neutrophils (Figure 4e), M0 macrophages (Figure 4f), M1 macrophages (Figure 4g), CD4 memory resting T cells (Figure 4h), and CD8 T cells (Figure 4i) in LGG, but negatively correlated with activated mast cells (Figure 4j) and M2 macrophages (Figure 4k), for all the above *P* values were less than 0.05. We also analyzed the common immune checkpoints, and the correlation between immune checkpoints and STAT3 expression. As shown in Figure 4l, in LGG, the common immune checkpoint proteins CD274 (also known as PD-L1), PDCD1 (also known as PD-1), CTLA4, LAG3, and TIGIT showed a significant positive correlation with STAT3 expression.

### 3.4 GSEA

**Figure 5:**
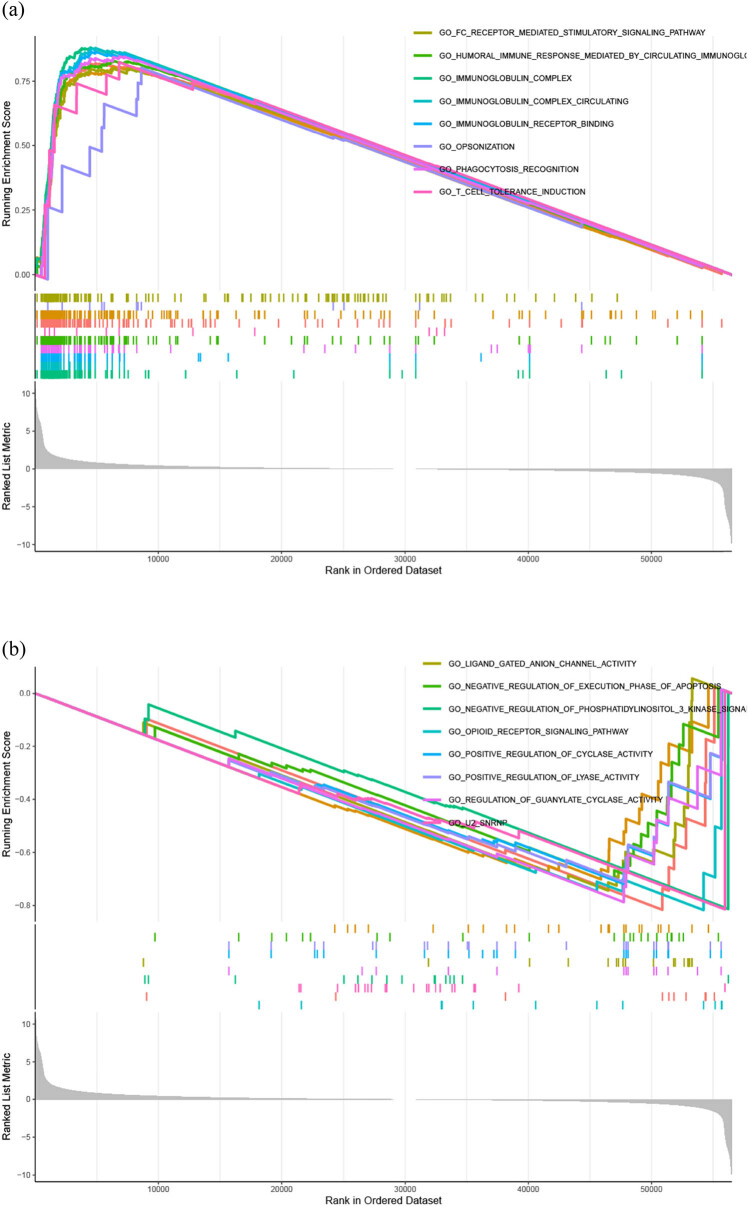
GSEA enrichment analysis: (a) first ten upregulated pathways and (b) first ten downregulated pathways.

Our GSEA identified 384 enrichment gene sets. According to the enrichment scores, the top ten upregulated pathways were mainly related to the immune response and included the following: immunoglobulin complex, immunoglobulin complex circulating, immunoglobulin receptor binding, phagocytosis recognition, humoral immune response mediated by circulating immunoglobulins, T cell tolerance induction, antigen binding, complement activation, opsonization, and Fc receptor mediated stimulatory signaling pathway (Figure 5a). The top ten downregulated pathways were mainly related to cell signal transduction and regulation of cell survival, proliferation, and metabolism and included the following: opioid receptor signaling pathway, amine binding, U2 snRNP, negative regulation of phosphatidylinositol 3 kinase (PI3K) signaling, regulation of guanylate cyclase activity, Gaba gated chloride ion channel activity, Gaba receptor complex, negative regulation of vascular associated smooth muscle cell migration, anchored component of synaptic vesicle membrane, and inhibitory extracellular ligand gated ion channel activity (Figure 5b).

### 3.5 Genetic alteration analysis

**Figure 6:**
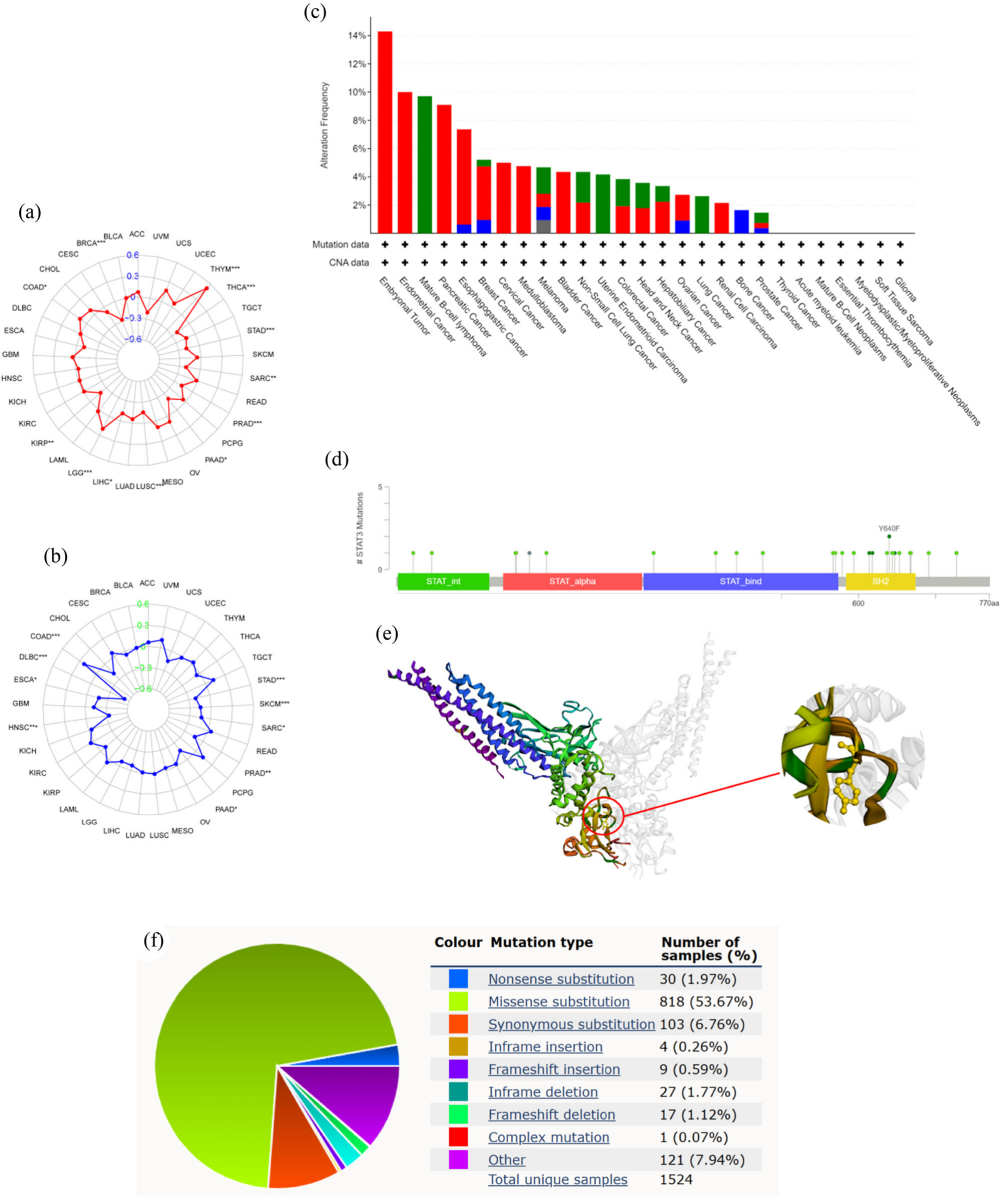

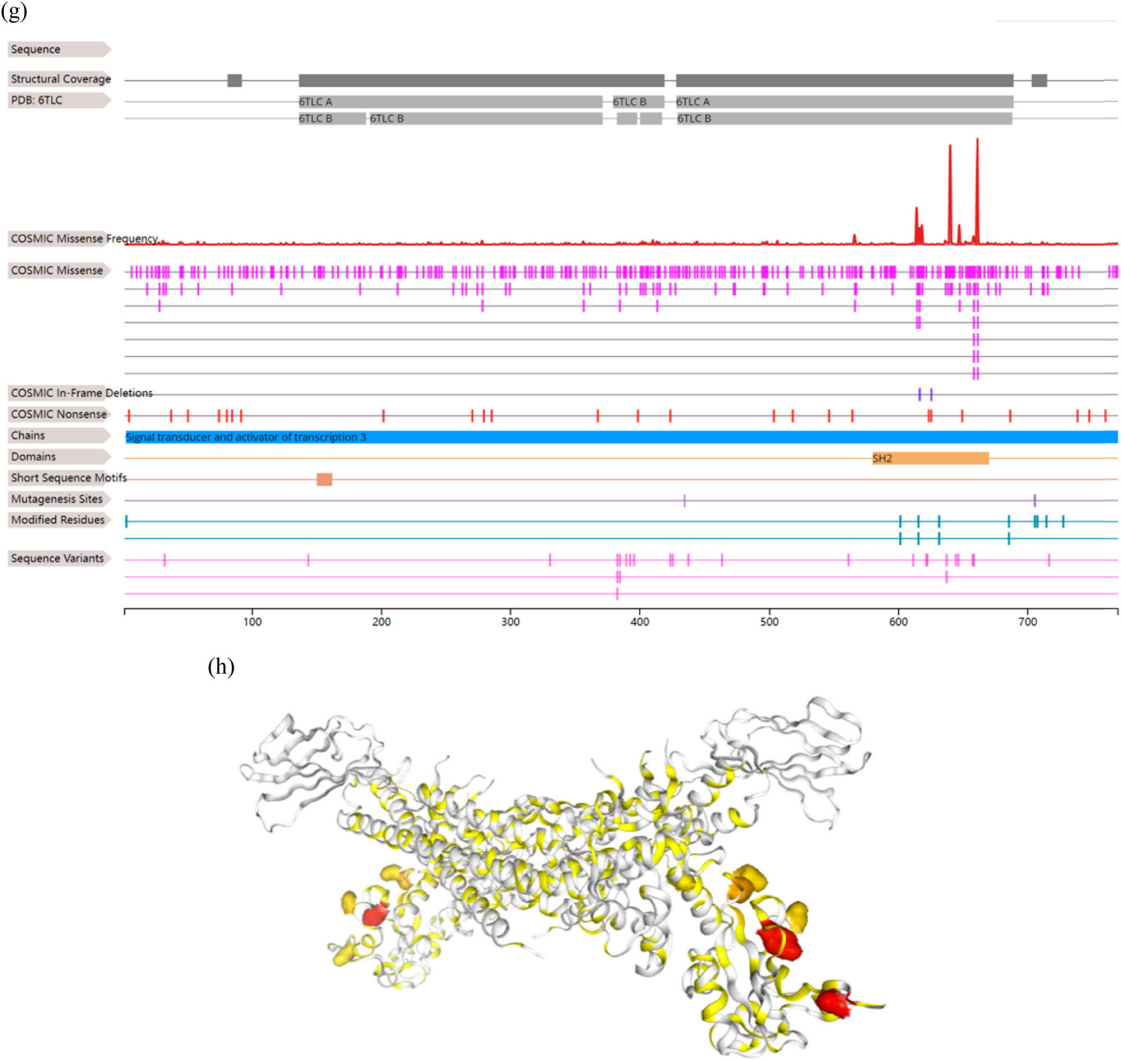
Genetic alteration analysis: (a) radar map shows the correlation between STAT3 expression and TMB, (b) radar map shows the correlation between STAT3 expression and MSI, (c) alteration frequency of ADAM12 in different tumors, (d) mutation lollipop chart provides information on the mutation sites and mutation types, (e) 3D structure of the STAT3 protein and the Y640F site, (f) an overview of the types of mutations observed that are curated by COSMIC, (g) protein sequence features of STAT3 protein curated by COSMIC, and (h) 3D structure and missense mutation frequency of STAT3 protein that are curated by COSMIC (the redder the color, the higher the missense mutation frequency).

As shown in the radar plot, the expression of STAT3 is negatively correlated with the TMB in BRCA, thyroid cancer, stomach adenocarcinoma (STAD), sarcoma (SARC), PRAD, pancreatic adenocarcinoma (PAAD), lung squamous cell carcinoma, liver hepatocellular carcinoma, and kidney renal papillary cell carcinoma, and positively correlated with thymoma (THYM), LGG, and COAD (Figure 6a, *P* < 0.05). The expression of STAT3 was negatively correlated with the MSI in STAD, SKCM, SARC, PRAD, PAAD, HNSC, esophageal carcinoma, and diffuse large B-cell lymphoma, but positively correlated with COAD (Figure 6b). Further analysis of the gene alterations in the CBioPortal database revealed, as shown in the total gene alteration histogram (Figure 6c), that embryonic tumors, endometrial carcinoma, and mature B-cell lymphoma are the top three cancers with the highest frequency of STAT3 alteration. In the “Mutations” module, the STAT3 mutation lollipop chart provides information on the mutation sites, and mutation types, as shown in Figure 6d, reveals that most of the STAT3 mutations occur in the SH2 domain, where both mutations in Y640F were “Missense”. The 3D structures of the STAT3 protein and the Y640F site are shown in Figure 6e. We further characterized the STAT3 gene mutation using the COSMIC database for STAT3 mutation analysis. The results of this analysis revealing the point mutations, copy number variation, overexpression or underexpression, and methylation of STAT3 in each group are shown in Table 2. The results in Table 2, which are sorted according to the point mutation frequency in descending order, show that vagina, penis, skin, hematopoietic, lymphoid, and liver tissues have the higher STAT3 mutation frequency. The “Missense substitution” of STAT3 accounted for the majority (51.88%) of mutations, as can be seen in the sector map of the mutation type (Figure 6f). The STAT3 protein sequence features map (Figure 6g) and its 3D structure map (Figure 6h) reveal that most of these missense substitutions are concentrated in the SH2 domain, which is consistent with the results of the analysis of the CBioPortal database.

**Table 2:** Distribution of mutations across the primary tissue types that are curated by COSMIC

	Point mutations		Copy number variation		Gene expression		Methylation	
	Mutated (%)	Tested	Variant (%)	Tested	Regulated (%) (over/under)	Tested	Diff. methylated (%)	Tested
Vagina	50	2	−	−	−	−	−	−
Penis	11.76	17	−	−	−	−	−	−
Skin	4.75	2,482	−	−	4.44/1.27	473	−	−
Hematopoietic and lymphoid	4.71	11,951	−	−	5.43/3.17	221	−	−
Liver	4.01	2,917	0.3	663	3.75/−	373	−	244
Endometrium	3.86	1,062	0.17	586	3.99/4.65	602	−	398
Vulva	3.33	30	−	−	−	−	−	−
Placenta	2.94	34	−	−	−	−	−	−
Prostate	2.48	3,102	0.21	949	2.01/0.4	498	−	−
Cervix	2.31	389	−	−	6.19/−	307	−	−
Urinary tract	2.26	1,285	0.25	399	2.45/−	408	−	−
Biliary tract	2.22	1,173	−	−	−	−	−	−
Large intestine	2.06	4,717	−	−	3.44/3.28	610	0.36	281
Upper aerodigestive tract	2.04	1,811	−	−	3.26/0.19	522	−	496
Ovary	1.92	1,460	0.29	684	1.5/0.38	266	−	−
Stomach	1.88	1,912	0.42	472	2.11/−	285	−	−
Pancreas	1.84	2,550	0.11	898	3.91/5.03	179	−	−
Meninges	1.52	198	−	−	−	−	−	−
Breast	1.45	5,445	0.27	1,492	4.35/2.17	1,104	−	707
NS	1.3	460	−	−	−	−	−	−
Esophagus	1.28	1,800	0.39	510	2.4/0.8	125	−	−
Salivary gland	1.12	267	−	−	−	−	−	−
Lung	1.07	5,619	0.4	1,006	4.32/1.37	1,019	−	717
Soft tissue	1.02	1,561	−	−	3.8/1.9	263	−	−
Small intestine	0.66	305	−	−		−	−	−
Thyroid	0.66	1,976	−	−	2.53/1.56	513	−	510
Kidney	0.56	2,870	0.1	995	3.67/3.83	600	2.53	513
Central nervous system	0.36	3,370	0.1	1,035	4.45/0.14	697	−	−
Adrenal gland	0.31	654	0.37	267	2.53/3.8	79	−	−
Autonomic ganglia	0.08	1,231	−	−	−	−	−	−
Bone	−	737	−	−	−	−	−	−
Eye	−	176	−	−	−	−	−	−
Fallopian tube	−	3	−	−	−	−	−	−
Gastrointestinal tract (site indeterminate)	−	67	−	−	−	−	−	−
Genital tract	−	126	−	−	−	−	−	−
Parathyroid	−	35	−	−	−	−	−	−
Perineum	−	1	−	−	−	−	−	−
Peritoneum	−	38	−	−	−	−	−	−
Pituitary	−	86	−	−	−	−	−	−
Pleura	−	356	1.15	87	−	−	−	−
Testis	−	458	−	−	−	−	−	−
Thymus	−	180	−	−	−	−	−	−
Uterine adnexa	−	4	−	−	−	−	−	−

## 4 Discussion

Increasing evidence shows that aberrant activation of STAT3 is involved in the proliferation and survival of tumor cells. The purpose of this study is to investigate the effects of genetic alterations of STAT3 and its expression on the development of cancer from the perspective of pan-cancer. First, we analyzed the differential expression between normal tissues and pan-cancer tissues, and found that the expression of STAT3 was different in different tumors and different tissues, and there were significant differences between most tumors and normal tissues. We also evaluated the effect of STAT3 expression on the prognosis of cancer patients by performing survival analysis, which revealed that, in LGG, the prognosis of OS and PFS in the STAT3 high expression group was worse than that in the low expression group. To some extent, this finding indicated that the high expression of STAT3 was related to the poor prognosis of gliomas. Therefore, we focused our study on the effect of STAT3 on gliomas. We further analyzed the glioma dataset downloaded from the CGGA database to verify the aforementioned results of STAT3 in glioma, and the results similarly suggested that the STAT3 high expression group had a poor prognosis. Additionally, univariate and multivariate regression analysis and ROC curve analysis indicated that STAT3 can be used as an independent prognostic factor of glioma with a certain degree of robustness. The importance of the IME in tumorigenesis and malignant progression is currently a hot research topic. Numerous studies have shown that the IME can promote the progression of cancer and lead to drug resistance, especially to cancer immunotherapy [34,35]. Therefore, we performed immune correlation analysis and GSEA to further investigate the mechanism by which activated STAT3 leads to poor cancer prognosis.

The immune correlation analysis found that the immune score and matrix score were higher in the group with high expression of STAT3. Further analysis of the infiltration of immune cell infiltration revealed that STAT3 was positively correlated with DCs, eosinophils, neutrophils, M0 macrophages, M1 macrophages, CD4 T cells, and CD8 T cells, and negatively correlated with activated mast cells and M2 macrophages. Previous studies have shown that abnormal STAT3 activation promotes the recruitment immune cells and impairs their function, resulting in immune escape of tumor cells [36]. First, aberrant activation of STAT3 in tumor cells plays an important role in the maturation of DCs. DCs are key antigen presenting cells of the immune system and play an important role in initiating the response of T cells to tumors, while immature DCs usually induce immune tolerance [37]. Overactivation of STAT3 in tumor cells can interfere with the antigen presentation process of DCs in various ways, such as decreasing the expression of BCL2 in DCs by inhibiting the expression of IL12 and TNF [38–40], and inhibiting the maturation of DCs and innate immunity by negatively regulating the expression of interferon gamma inducible protein 10 and CC chemokine ligand 5 [41]. Moreover, since immature DCs cannot activate antigen-specific CD8+ T cells, the antitumor effect of CD8+ T cells will be decreased accordingly. In addition, other studies have found that STAT3 plays a major role in the expansion of regulatory T cells, and regulatory T cells can promote tumor progression by inhibiting the antitumor immune response mediated by TH1 CD4+ T and CD8+ T cells [40,42,43]. Furthermore, overactivation of STAT3 can promote tumor progression by inducing the polarization of type M2 macrophages and the expression of CD274 [44]. In summary, it is clear that overexpression of STAT3 can regulate in various ways the tumor IME, which besides generally promoting tumor progression, is related to poor prognosis, and is consistent with our findings. Through GSEA, we found that many immune response pathways are activated in the STAT3 high expression group, such as antigen–antibody binding, immunoglobulin complex formation, phagocytosis recognition, complement activation, etc. It is worth noting that among the downregulated pathway, the “negative regulation of PI3K signal transduction pathway” is downregulated. PI3K is a major regulatory factor of cancer, which can affect the progression of cancer by affecting the growth, proliferation, survival, and angiogenesis of tumor cells [45−51]. The results of this study suggest that the negative regulation of PI3K signaling is downregulated in the group with high expression of STAT3. Previous studies have revealed that there is a certain correlation between STAT3 and the PI3K signaling pathway. First, Hart et al. identified the dependent transcription between PI3K and STAT3 by analyzing stable isotope labeling with amino acids in cell culture of PI3K transformed cells [52]. Subsequently, Hart et al. further studied the STAT3 and PI3K pathways and reported the following findings: (1) The p110 α-H1047R mutant transformed cells of PI3K showed increased tyrosine phosphorylation of STAT3. (2) The dominant-negative mutation of STAT3 interferes with PI3K-induced tumorigenesis. (3) GDC-0941, a specific inhibitor of PI3K, can reduce the phosphorylation level of STAT3. (4) In some human tumor cell lines, the enhanced phosphorylation of STAT3 is inhibited by PI3K and Tec kinase inhibitors. In summary, the study of the regulatory relationship between PI3K and STAT3 is of great significance to understand the development of cancer, and the inhibition of STAT3 expression may represent a breakthrough in the treatment of human tumors [53,54]. In order to develop new approaches to target STAT3 inhibitors, we also performed genetic alteration analysis. The TMB and MSI have been considered as predictive biomarkers of immune checkpoint blocking responses. Our study revealed that the expression of STAT3 is associated with TMB and MSI in many tumors, which suggests the possibility that, to some extent, STAT3 may serve as an immune checkpoint in these tumors [54−56]. By examining the impact of genetic alterations in STAT3, we found that there are alterations in STAT3 in many tumors. In the mutational analysis of STAT3, our analysis showed that missense mutations in STAT3 account for the vast majority of mutations, and STAT3 mutations mostly occurred in the SH2 domain. The SH2 domain is the most conserved STAT domain, which drives transcription by binding to a specific phosphotyrosine motif that is essential for molecular activation and nuclear accumulation of phosphorylated STAT dimers. Even slight changes in the electronic or stereo structure of the SH2 domain can significantly change the activity of STAT3 [13,57,58]. To date, using high-throughput screening and a structure-based virtual screening system, a variety of small molecular peptides of STAT3 directly targeting the SH2 domain of STAT3 have been reported, which can also significantly change the activity of STAT3 [53]. For example, PY*LKTK (where Y* is the phosphorylated tyrosine) [59], S3I-M2001 [60], S3I-1757 [61], curcumin-proline [62], cryptotashinone [63], STA-21 [64], Stattic [65], S3I-201 [66], SD-36 [57], etc.

Undoubtedly, this study has certain limitations, including the following: this study is based on the analysis of multiple databases, and there are some differences in statistical analysis methods among different databases. More importantly, this is only a bioinformatics analysis study, and more genetic, experimental studies, and multicenter clinical studies are needed to verify the above inferences for more effective clinical application.

## 5 Conclusion

Overexpression of STAT3 promotes the growth and development of tumor cells by regulating the IME, which is significantly related to poor prognosis in cancer patients. Therefore, targeted inhibition of STAT3 expression or activity may have important research value for the development of combined immunotherapy and targeted therapy approaches for the treatment of cancer patients.
